# Prevention of Sudden Cardiac Death

**Published:** 2011-07-03

**Authors:** Johnson Francis

**Affiliations:** 1 Professor of Cardiology, KMCT Heart Institute & Research Centre, KMCT Medical College, Calicut, Kerala, India; 2 Senior Consultant Cardiologist, Baby Memorial Hospital, Calicut, Kerala, India

**Keywords:** sudden cardiac death, SCD, prevention

Sudden cardiac death (SCD) due ventricular tachyarrhythmias is an important cause of mortality world wide. While the majority of these deaths occur in those with structural heart disease, a small percentage (about 4%) can occur in those with structurally normal heart as well [1]. Such primary electrical disorders of the heart which cause SCD are now called as channelopathies as they involve the dysfunction (either a gain or a loss of function) of cardiac ion channels.

In this issue of the journal, Madhavan SR et al [2] present epidemiological data on SCD in South India based on a prospective mortality surveillance conducted in 45 villages with 180,162 subjects. They noted that SCD contributed to about half of the cardiovascular deaths in rural South India. SCD contributed to 17% of the total deaths excluding accidental deaths. Hypertension, diabetes mellitus and a history of myocardial infarction / coronary artery disease were noted to be the significant associations.

Sudden cardiac death can be an initial presentation or preceded by symptoms related to arrhythmias or hemodynamically significant cardiovascular disorders. Sometimes evaluation is called for due to family history of sudden cardiac death. In any case, the evaluation begins with a detailed history of clinical details, family history if present and a clinical evaluation to look for potential conditions which can cause SCD. This will be followed by the assessment of a baseline electrocardiogram and other investigations as deemed necessary. An echocardiogram is useful in documenting structural heart diseases prone for SCD like severe aortic stenosis and hypertrophic cardiomyopathy. Stress testing for myocardial ischemia or inducible arrhythmia; Holter monitoring, loop monitoring and implantable loop recorders for documenting siginificant arrhythmia and finally genetic testing in case of suspected inherited arrhythmias would be other important investigations to be undertaken in this setting.

Prevention of SCD in heart failure and left ventricular dysfunction assumes great importance as this is one of the largest groups in the whole arena of sudden cardiac death. Rather than pump failure, sudden cardiac death is the most common mode of death in persons with left ventricular dysfunction, more so in those with mild and moderate left ventricular dysfunction. Pump failure deaths definitely increase in those with severe left ventricular failure. Implantable defibrillators have been shown to be the most important way of prevention of SCD in this subset of patients by various large clinical trials like Multicenter Automatic Defibrillator Implantation Trial (MADIT), Multicenter Unsustained Tachycardia Trial (MUSTT), and MADIT-II [3]. Optimal treatment of heart failure with medications will also go a long way in reducing the symptoms and possibility of sudden cardiac death in this group of patients.

At the community level, measures to improve the coronary risk factors can bring down the incidence of coronary artery disease as it is a major contributor to SCD. Another important aspect at the community level is the implementation of emergency medical services which can provide immediate medical care at the onset of symptoms of diseases which can lead to sudden cardiac death.

Even though implantation of cardioverter-defibrillator is finally the answer in prevention of SCD in most inherited arrhythmias, several other interventions have been found useful. These include beta blockers in catecholaminergic polymorphic ventricular tachycardia and many subtypes of congenital long QT syndromes, quinidine in Brugada syndrome [3] and left cardiac sympathetic denervation in congenital long QT syndrome and catecholaminergic polymorphic ventricular tachycardia [4]. Interestingly, left cardiac sympathetic denervation has also been shown to reduce the incidence of sudden cardiac death among certain subgroups of post myocardial infarction patients at high risk for SCD [5].
